# Supplementary material for: The Tubulin Superfamily in Apicomplexan Parasites
Source: Microorganisms. 2023 Mar 9;11(3):706. doi: 10.3390/microorganisms11030706 (PMC10056924; doi:10.3390/microorganisms11030706)
Supplement: Supplementary file 1 [file microorganisms-11-00706-s001.zip › Supplemental Table S1.pdf]

**Table S1:** Uniprot accession numbers for tubulins		
	Tetrahymena	*Chlamydomonas*
α-tubulin	P41351	P09204<br>P09205
β-tubulin	P41352	P04690
γ-tubulin	O00849	Q39582
δ-tubulin	A4VDW7<br>I7MJY7	O22416
ε-tubulin	Q22YZ9	Q8LRU3<br>A0A2K3DX56
