# Supplementary material for: The Tubulin Superfamily in Apicomplexan Parasites
Source: Microorganisms. 2023 Mar 9;11(3):706. doi: 10.3390/microorganisms11030706 (PMC10056924; doi:10.3390/microorganisms11030706)
Supplement: Supplementary file 1 [file microorganisms-11-00706-s001.zip › Supplemental Table S2 P. falciparum transcripts.pdf]

Supplemental Table S2: *P. falciparum* transcripts

TPMs	Male gametocyte	Female gametocyte	Asexuals Blood Sateg	Midgut oocysts	Salivary gland sporozoites	Rings	Trophozoites	Late trophozoites	Schizonts	Gametocytes II	Gametocytes V	Ookinetes	Sporozoites	Oocysts	Rings
PF3D7_0903700	117.12	823.04	177.25	313.23	27.72	22.74	44.85	384.81	289.76	119.99	198.01	803.96	15.68	510.09	34.84
PF3D7_0422300	8193.89	164.59	268.32	28.02	1.29	57.29	19.6	199.73	31.94	218.46	277.27	318.58	4.41	44.86	35.99
PF3D7_1008700	586.64	270.26	68.55	229.9	21.01	33.02	39.26	637.59	248.09	237.32	201.96	312.03	28.76	918.79	65.72
PF3D7_0803700	246.66	83.7	33.59	41.33	5.2	13.3	7.24	26.45	4.84	16.41	42.2	93.24	6.14	60.78	5.26
PF3D7_0933800	275.03	8.27	22.85	2.09	1.46	5.06	0.38	2.03	0.28	0.62	5.64	0	1.46	11.65	0.85
PF3D7_1475700	364.51	8.11	35.89	0.19	4.97	7.16	0.71	0.57	0.2	0.41	6.03	0	4.04	1.47	3.26

Plasmodium falciparum transcriptomics data, sense, unique only, extracted from the following paper. Datasets labeled in red type are represented in Figure 5.

Lasonder, E.; Rijpma, S. R.; van Schaijk, B. C.; Hoeijmakers, W. A.; Kensche, P. R.; Gresnigt, M. S.; Italiaander, A.; Vos, M. W.; Woestenenk, R.; Bousema, T.; Mair, G. R.; Khan, S. M.; Janse, C. J.; Bartfai, R.; Sauerwein, R. W., Integrated transcriptomic and proteomic analyses of *P. falciparum* gametocytes: molecular insight into sex-specific processes and translational repression. Nucleic Acids Res 2016, 44, (13), 6087-101.

Gomez-Diaz, E.; Yerbanga, R. S.; Lefevre, T.; Cohuet, A.; Rowley, M. J.; Ouedraogo, J. B.; Corces, V. G., Epigenetic regulation of *Plasmodium falciparum* clonally variant gene expression during development in *Anopheles gambiae*. Sci Rep 2017, 7, 40655.

Lopez-Barragan, M. J.; Lemieux, J.; Quinones, M.; Williamson, K. C.; Molina-Cruz, A.; Cui, K.; Barillas-Mury, C.; Zhao, K.; Su, X. Z., Directional gene expression and antisense transcripts in sexual and asexual stages of *Plasmodium falciparum*. BMC Genomics 2011, 12, 587.

Zanghi, G.; Vembar, S. S.; Baumgarten, S.; Ding, S.; Guizetti, J.; Bryant, J. M.; Mattei, D.; Jensen, A. T. R.; Renia, L.; Goh, Y. S.; Sauerwein, R.; Hermsen, C. C.; Franetich, J. F.; Bordessoulles, M.; Silvie, O.; Soulard, V.; Scatton, O.; Chen, P.; Mecheri, S.; Mazier, D.; Scherf, A., A Specific PfEMP1 Is Expressed in *P. falciparum* Sporozoites and Plays a Role in Hepatocyte Infection. Cell Rep 2018, 22, (11), 2951-2963.
