# Supplementary material for: The Tubulin Superfamily in Apicomplexan Parasites
Source: Microorganisms. 2023 Mar 9;11(3):706. doi: 10.3390/microorganisms11030706 (PMC10056924; doi:10.3390/microorganisms11030706)
Supplement: Supplementary file 1 [file microorganisms-11-00706-s001.zip › Supplemental Table S3 T. gondii transcripts.pdf]

Supplemental Table S3: *T. gondii* transcripts

Normalized counts	Tachyzoites	Bradyzoites	EES1	EES2	EES3	EES4	EES5	Oocysts unsporulated	Oocysts sporulating	Oocysts sporulated
TGME49_316400	33692	19574	25920	28596	27241	30082	36827	86434	125514	3777
TGME49_231770	1	0	101	313	585	951	3905	50882	38891	821
TGME49_231400	0	52	135	270	306	252	360	1210	835	375
TGME49_266960	18227	12173	15839	17413	14576	15103	18623	115220	123901	7880
TGME49_221620	5846	1865	4199	7699	9072	7506	12320	18053	23603	2311
TGME49_212240	42	117	125	331	679	913	2172	375	499	671
TGME49_226870	696	283	697	525	423	448	454	103	114	182
TGME49_207600	343	1516	139	184	165	204	272	67	92	41
TGME49_275870	334	397	105	163	194	269	388	32	412	2878

Toxoplasma gondii transcriptomics data (normalized counts)

Data from C. Ramakrishnan and A. Hehl (unpublished, deposited in ToxoDB) and published in Ramakrishnan, C.; Maier, S.; Walker, R. A.; Rehrauer, H.; Joekel, D. E.; Winiger, R. R.; Basso, W. U.; Grigg, M. E.; Hehl, A. B.; Deplazes, P.; Smith, N. C., An experimental genetically attenuated live vaccine to prevent transmission of *Toxoplasma gondii* by cats. Sci Rep 2019, 9, (1), 1474.

Highlighted columns represent data graphed in figure 5.
