# Supplementary figures and images for: The Tubulin Superfamily in Apicomplexan Parasites
Source: Microorganisms. 2023 Mar 9;11(3):706. doi: 10.3390/microorganisms11030706 (PMC10056924; doi:10.3390/microorganisms11030706)

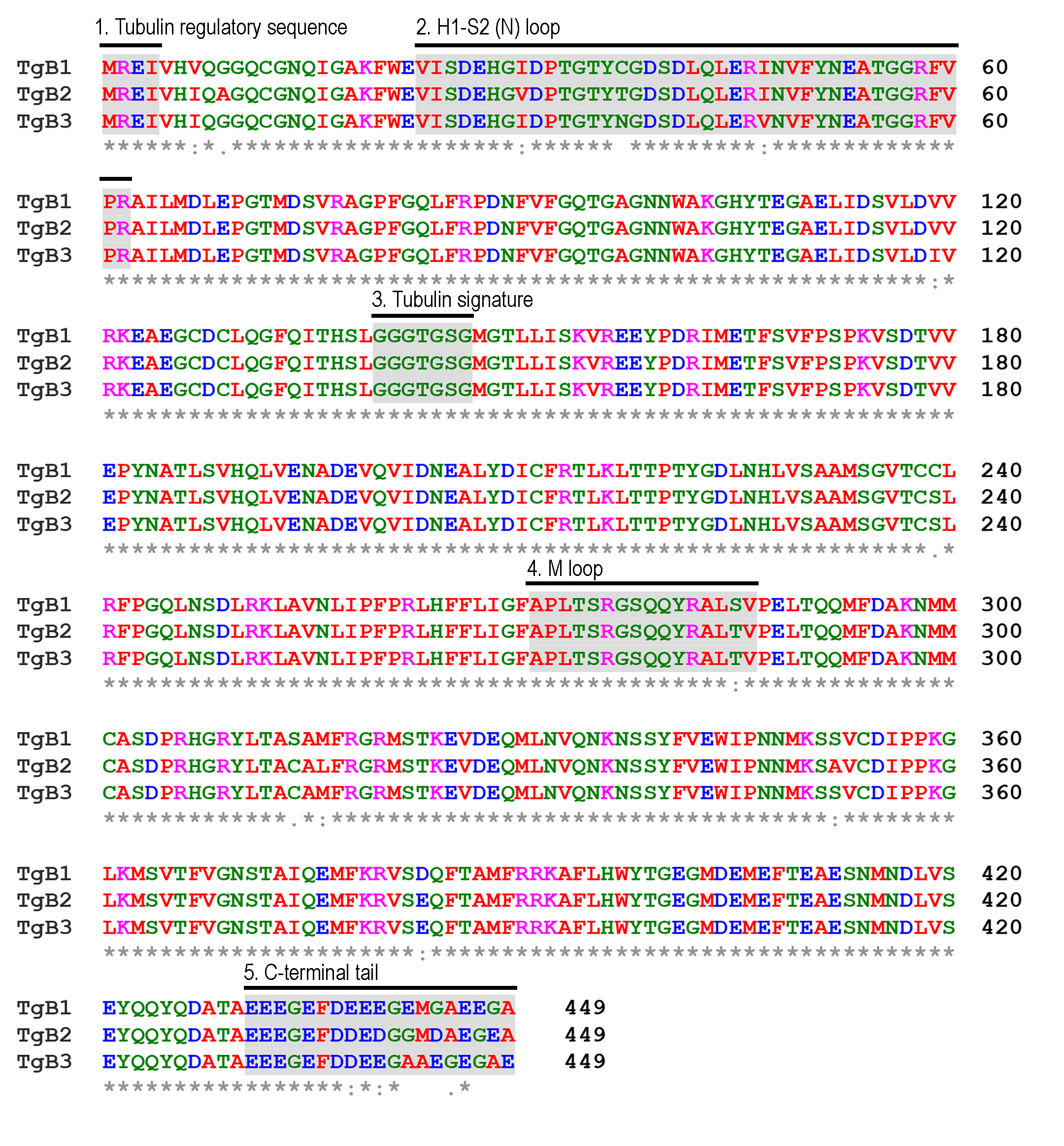

Supplement: Supplementary file 1 [file microorganisms-11-00706-s001.zip › Suppl Figure S1 beta tubulins.tif]
